# Mean platelet volume, platelet count, and neutrophil/lymphocyte ratio in drug-naïve patients with schizophrenia: a cross-sectional study

**DOI:** 10.3389/fpsyt.2023.1150235

**Published:** 2023-05-09

**Authors:** Héctor Cabello-Rangel, Marisol Basurto-Morales, Elizabeth Botello-Aceves, Osiris Pazarán-Galicia

**Affiliations:** ^1^Research Department, Hospital Psiquiátrico “Fray Bernardino Álvarez”, Ciudad de México, Mexico; ^2^Clinic Laboratory Department, Hospital Psiquiátrico Infantil “Juan N. Navarro”, Ciudad de México, Mexico; ^3^Medical Student, Universidad Quetzalcóatl, Irapuato, Guanajuato, Mexico

**Keywords:** platelets, mean platelet volume, schizophrenia, neutrophil/lymphocyte ratio, psychosis

## Abstract

**Introduction:**

Mean platelet volume (MPV), platelet count, and neutrophil/lymphocyte ratio (NLR) have been proposed to be biomarkers of the chronic inflammatory process in schizophrenia and indicative of increased cardiovascular risk.

**Objective:**

To describe MPV, total platelet count (PLT), and NLR between healthy controls and patients with schizophrenia to determine the correlation between these parameters and the duration of untreated psychosis (DUP).

**Methods:**

In a retrospective cross-sectional study, we included 175 patients with schizophrenia who had never received psychiatric treatment, and who had undergone blood biometry and blood chemistry within 24 h of admission. Laboratory studies were determined by the impedance method on Coulter ac-T 5 diff hematological equipment.

**Results:**

Mean platelet volume levels in patients with schizophrenia were higher than in healthy controls but not statistically significant. The receiver operating characteristic curve for this parameter shows that the optimal cutoff point of agreement was 8.95 fL, with sensitivity and specificity for schizophrenia of 52% and 67%, respectively, and the area under the curve (AUC) was 0.580 (*p* = 0.079). DUP had no significant correlation with the blood parameters analyzed.

**Conclusion:**

The results partially support the hypothesis that MPV, platelet count, and NLR is related to schizophrenia, and further research is needed to establish whether there is an underlying chronic inflammatory process.

## Introduction

High mean platelet volume (MPV) can be considered and used as a prognostic factor in numerous inflammatory diseases, for example, for cardiac infarction in patients with coronary artery disease, mortality from myocardial infarction, cerebrovascular ischemia, respiratory diseases, or cancer (1). It has also been reported that high MPV increases the risk of death from acute myocardial infarction compared with those with normal MPV (2, 3).

The potential mechanism of action by which high MPV, increases cardiovascular risk is that larger platelets have more α-granules, which release prothrombotic factors (P-selectin, thromboxane A2, and B2; ADP and serotonin), which increases the level of platelet aggregation through glycoprotein IIb–IIIa receptors, with consequent vasoconstriction, inflammation, and induction of thrombosis (3).

Experimental studies in animal models with chronic inflammatory states indicated increased mean platelet volume and P-selectin overexpression (4). It has been demonstrated that platelets release cytokines and chemokines upon activation, mainly interleukin-6 (IL-6), interleukin-8 (IL-8), and chemokines (CCL) (5, 6). Platelets have pro-inflammatory action through multiple receptors including major histocompatibility complex-1 (MHC-1), toll-like receptors (TLR), receptors for advanced glycation end products (RAGE), CD40 ligands, and P-selectin (7).

Controversy exists about the involvement of MPV in the inflammatory process. Some studies have found no association between MPV and the inflammation process (8). However, it has been demonstrated in multiple pathologies that platelet size, and, therefore, its activity, is closely related to the severity of the inflammatory process. In the case of acute inflammatory events, platelet activation and consumption tend to increase, therefore, MPV is decreased. On the other hand, in more chronic processes, platelet reactivity reaches an equilibrium and MPV tends to increase (9).

In mental disorders, a significant increase in MPV value has been reported in patients with schizophrenia treated with atypical antipsychotics compared to patients with schizophrenia without antipsychotic treatment. However, a much higher MPV value has also been observed in patients with schizophrenia without antipsychotic treatment compared with a group of healthy subjects (10).

Another study described an increase in MPV in patients with schizophrenia on treatment with typical or atypical antipsychotics compared to a control group the same study assessed platelet counts in both groups and identified a decreased value in the schizophrenia group compared to the healthy group. In addition, the schizophrenia group had a higher body mass index than the control group, which could be related to the increase in MPV (11).

A significantly lower MPV value has been documented in patients with the first episode of schizophrenia and mania compared to the control group, and later studies have described an increase in MPV and PLT in patients with schizophrenia and TB; however, these were performed after the first episode and in patients under treatment with antipsychotics, which is why these values could be found to be increased (12).

Another indicator of systemic inflammation is the neutrophil/lymphocyte ratio, established as an indicator of strong systemic inflammation associated with mortality and adverse cardiac events, a study that analyzed the combination of high neutrophil/lymphocyte ratio and high mean platelet volume concluded that it independently predicts major adverse cardiovascular events in patients with coronary syndrome (13). Thus, both MPV and NLR can be used as biomarkers of inflammation as predictors of adverse arterial events due to atheroma plaque formation (14).

It has been shown that platelet–lymphocyte interaction triggers a series of events in the endothelial wall that stimulates atherogenesis by stimulation of molecular adhesion and active expression of leukocyte cytokines that cause adhesion to the arterial endothelium and promotion of atherosclerotic lesions, in addition to platelet activation and release of atherogenic chemokines that stimulate the entry of monocytes and neutrophils into the injured tissue (15).

Similarly, high levels of NLR and MLR have been reported in patients with schizophrenia relative to healthy controls and patients with depression (16). However, it has been pointed out that there are substantial deficiencies in the available research, for example, small sample sizes, lack of information on medication, lack of association with symptomatology, as well as methodological standards and comparability of blood sampling procedures, which may lead to biases (17).

In this regard, it has been speculated that the use of atypical antipsychotics may cause increased platelet metabolic and enzymatic activity (18), which may be due to alteration of platelet membrane lipids, surface receptors, intracellular messengers, or signal transduction, however, the mechanism is not defined (19).

Our objective was to describe and compare MPV, total platelet count, and neutrophil/lymphocyte ratio (NLR) between healthy controls and drug-naïve patients with schizophrenia as well as, to determine if there is a correlation between these blood parameters and the duration of untreated symptoms (DUP). The DUP was defined as the time between the onset of psychotic symptoms and the initiation of antipsychotic treatment (20).

## Materials and methods

This is an observational, cross-sectional, retrospective study. Clinical records of users attended in the period between January 2016 and October 2018 in a public psychiatric hospital in Mexico City were included.

The sampling was consecutive, using a database of first-time patients who met the following inclusion criteria: both sexes, aged between 18 and 50 years, with a diagnosis of schizophrenia made by a certified psychiatrist based on the diagnostic criteria of the International Classification of Diseases (ICD-10), drug-naïve patients who had undergone blood biometry and blood chemistry within the first 24 h of admission.

**Figure 1:**
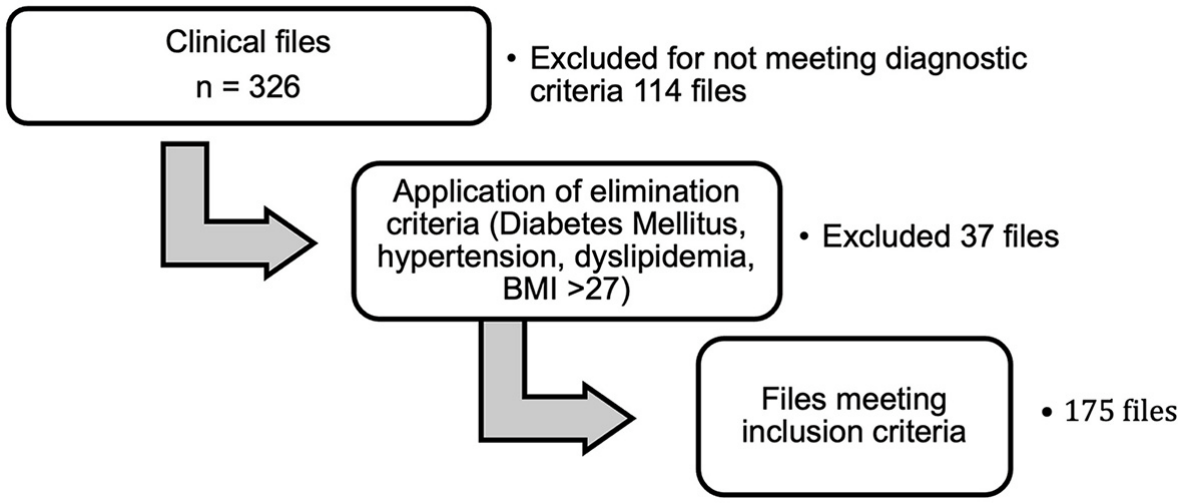
Clinical file analysis process.

Exclusion criteria were files with the results of blood biometry with total leukocytes >11,000 cells/ml, users who were pregnant or breastfeeding, users with chronic degenerative diseases (diabetes mellitus, arterial hypertension, cardiovascular disease, and dyslipidemia), hematological disorders, cancer, autoimmune disorders, and use of anticoagulants (Figure 1).

### Blood sample

Blood samples were collected in violet-capped tubes with ethylenediaminetetraacetic acid (EDTA) anticoagulant in the first 24 h of admission at 08:00 a.m. during the laboratory routine, with fasting from 8 to 12 h. The mean platelet volume, platelets, and leukocytes were determined by the impedance method in Coulter ac-T 5diff hematological equipment (Beckman equipment). The reference ranges were as follows: leukocytes 4–10 × 103 cells/ml, mean platelet volume 6–10 fL, and platelets 150–450 × 103 cells/ml. All samples were processed within 1 h of collection.

### Clinical data

Each clinical record was analyzed to determine the age of the symptom and the duration of untreated psychosis (months) defined as the time between the onset of psychotic symptoms and initiation of antipsychotic treatment. They were analyzed to confirm that they had never received antipsychotic or other psychotropic treatment.

### Controls

Records from the GWlab database software of subjects aged between 18 and 50 years, men and women, who had laboratory studies, performed between the years 2016 and 2018. Studies of those subjects were taken at 08:00 a.m. during routine laboratory workup. Those with blood biometry and blood chemistry were included. Subjects with a diagnosis of type-II diabetes mellitus, pregnancy, cancer, hematological disorders, dyslipidemias, cardiovascular, hypertension, or alcohol dependence were excluded. Records with abnormal parameters in blood biometry and blood chemistry were also excluded.

### Data analysis

A descriptive statistical analysis was performed for quantitative variables and the mean and standard deviation (SD) were obtained. The neutrophil/lymphocyte ratio (NLR) was calculated by a simple arithmetic operation. The Kolmogorov–Smirnov test was used to determine the normality of distribution. A comparison of variables between groups was performed using the Mann–Whitney *U-*test. To determine the sensitivity and specificity of potential biomarkers, the receiver operating characteristic (ROC) curve for MPVs, platelets, and NLRs was constructed, subsequently, the X2 was estimated, and a statistical significance level of *p* < 0.05 was accepted. The association of variables was performed by calculating the spearman “rho” correlation coefficient and its 95% confidence interval. The analysis was performed using a Statistical Package for the Social Sciences (SPSS) version 26.0 for Windows.

### Ethical considerations

The protocol was approved by the Research Committee (registration 925, 2021) and the Research Ethics Committee (16 January 2022) of the “Fray Bernardino Álvarez” Psychiatric Hospital. We used clinical and laboratory records of patients and controls seen between January 2016 and October 2018.

## Results

A total of 175 files of patients with schizophrenia (mean age: 29.06 ± 10.1) and 52 controls (mean age: 33.85 ± 10.9) were assessed. The group of patients with schizophrenia included 132 men and 43 women; the group of controls comprised 14 men and 38 women. The mean duration of untreated psychosis was 1.04 ± 3.82 years and the mean age of symptom onset was 24.07 ± 8.05 years.

The distribution of the sample was abnormal either for the controls or for the group of patients with schizophrenia, except for the platelet count which was normal for both. The Mann–Whitney *U*-test showed no significant differences between the groups, i.e., the null hypothesis is accepted, except for the platelet count, which did reveal statistically significant differences (Table 1).

**Table 1:** Comparison of blood parameters between schizophrenia and control group.

		**Mean**	**Confidence interval**	**Median**	**Variance**	**Minimum**	**Maximum**	**Kolmogorov-Smirnov test**	**Mann U Whitney test**		
		**(SD)**	**(95%)**						**U**	**Z**	***P***
Leukocytes	SZ	6.61 ± 1.54	6.381–6.841	6.4	2.38	3.1	11	0.085^*^	4.11	−1.115	0.265
	Control	6.94 ± 1.69	6.469–7.408	6.6	2.84	3.7	10.8	0.106			
Neutrophils	SZ	3.78 ± 1.29	3.591–3.975	3.62	1.65	1.65	7.61	0.092^*^	4.097	−1.145	0.252
	Control	4.01 ± 1.30	3.6456–4.369	3.74	1.69	1.65	6.86	0.102			
Lymphocytes	SZ	2.11 ± 0.54	2.031–2.191	2.09	0.29	0.72	3.93	0.062	4.757	0.433	0.665
	Control	2.11 ± 0.66	1.924–2.294	2.08	0.44	1.09	5.49	0.151			
Platelets	SZ	242.69 ± 56.9	234.1–251.1	234	3,237.77	124	449	0.079^*^	2.619	−4.683	0.000
	Control	295.8 ± 74.7	275.0–316.6	299.5	5,589.43	149	449	0.127^*^			
MPV	SZ	9.02 ± 0.95	8.87–9.16	9	0.91	6.9	11.9	0.049	5.281	1.76	0.078
	Control	8.78 ± 0.79	8.55–8.99	8.6	0.63	7	10.5	0.126^*^			
NLR	SZ	1.91 ± 0.87	1.78–2.04	1.69	0.76	0.76	4.91	0.13	3.95	−1.49	0.14
	Control	1.99 ± 0.71	1.79–2.19	1.86	0.50	0.61	4.26	0.11			

^*^p < 0.05. MPV, mean platelet volume; NLR, neutrophil/lymphocyte ratio; SZ, schizophrenia.

**Figure 2:**
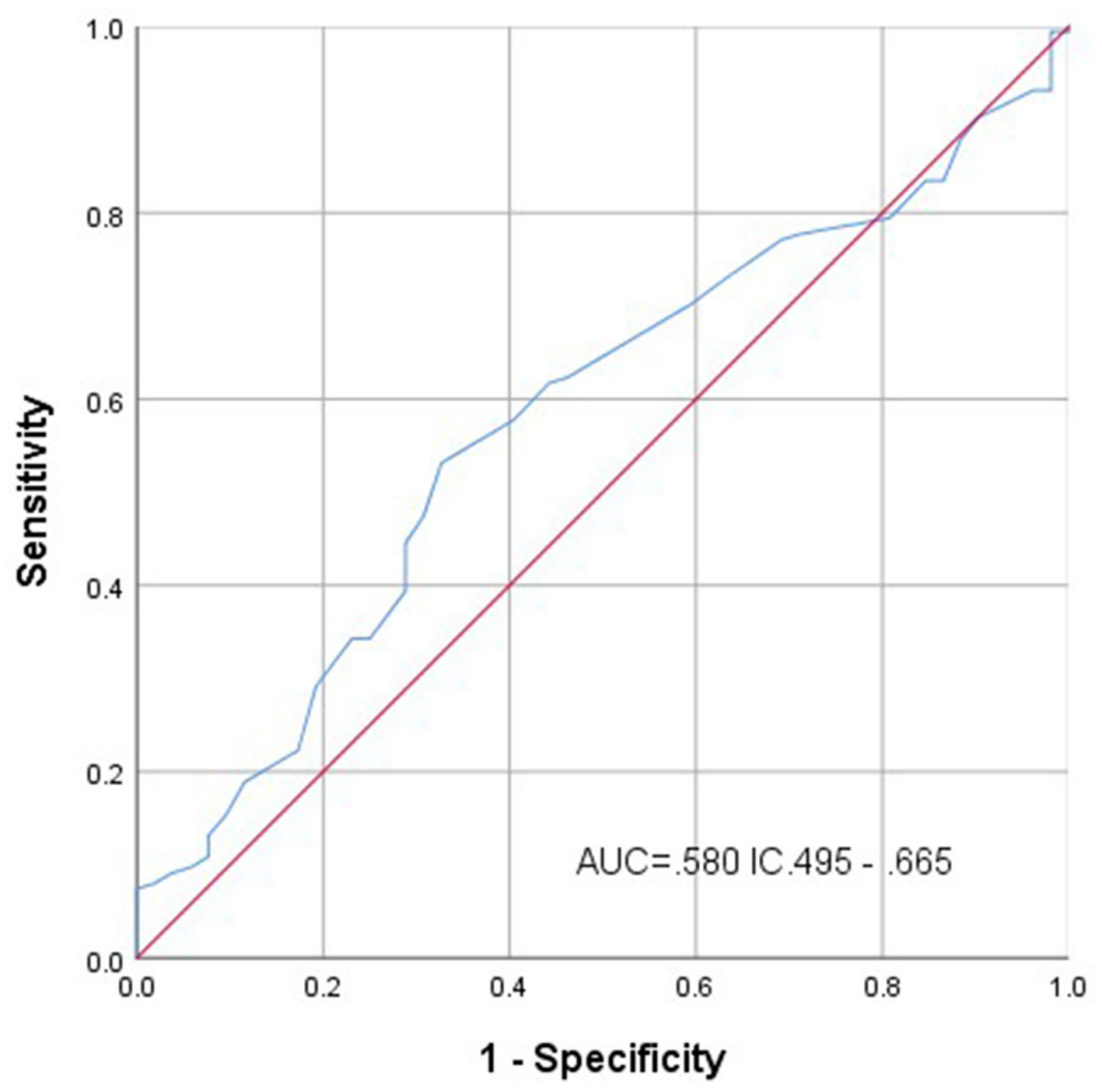
Receiver operating characteristic curve for the mean platelet volume variable.

The mean MPV of the group of patients with schizophrenia was higher with respect to the control group, with an alpha value close to a statistical significance (*p* = 0.078). The ROC curve for this parameter shows that the optimal cutoff point of agreement was 8.95 fL, with sensitivity and specificity for schizophrenia of 52 and 67%, respectively, and the area under the curve (AUC) was 0.580 (*p* = 0.079) (Figure 2).

The ROC curve for platelet count showed that the AUC was 0.283 CI 0.204–0.368 (*p* = 0.000); and for NLR, the AUC displayed no significative result. Pearson's X2 test, the MPV cutoff point was determined as 8.9 fL, it showed a value of 6.526 (*p* = 0.011) and Fisher's exact test of *p* = 008.

A positive and significant correlation was observed between the age of symptom onset and NLR (*p* = 0.006); platelet count correlated positively and significantly with leukocytes (*p* = 0.001). Duration of untreated psychosis had no significant correlation with the blood parameters analyzed (Table 2).

**Table 2:** Correlation between blood parameters and clinical variables.

	**ASO**	**DUP**	**Leukocytes**	**Platelets**	**MPV**	**NLR**
ASO	1	0.010	0.129	−0.032	0.126	0.208^**^
DUP	0.010	1	−0.109	0.086	0.115	−0.136
Leukocytes	0.129	−0.109	1	0.222^**^	−0.008	0.451^**^
Platelets	−0.032	0.086	0.222^**^	1	−0.465^**^	0.085
MPV	0.126	0.115	−0.008	−0.465^**^	1	−0.057
NLR	0.208^**^	−0.136	0.451^**^	0.085	−0.057	1

ASO, age of symptoms onset; DUP, duration of untreated psychosis; MPV, mean platelet volume; NLR, neutrophil/lymphocyte ratio. ^**^Correlation is significant at the 0.01 level (bilateral).

## Discussion

We analyzed 175 subjects who had never received psychopharmacological treatment in whom we measured the blood parameters, MPV, platelet count, and NLR to know if they can be considered biomarker tests for schizophrenia. The results show that platelet count is lower among patients with schizophrenia vs. healthy controls with statistically significant differences (*p* = 0.000), which is contrary to the hypothesis that elevated platelets may indicate an increased risk for developing cardiovascular disease in these patients. This finding coincides with another similar study that found no difference between the control group and platelet count but did report an inverse relationship between serotonin concentration and duration of illness (21).

We found that MPV was elevated with respect to controls, but it was not possible to demonstrate significant differences and according to ROC analysis, the sensitivity is low. Balcioglu and Kirlioglu also found high MPV and its correlation with the duration of illness and their regression analysis showed predictive value for a diagnosis of schizophrenia (22). Elevation of MPV is not indicative of an inflammatory process but has been observed in cases of thrombocytopenia with neutrophilia and leukocytosis in acute inflammation (6, 8). However, another study reports MPV low in patients vs. healthy controls (12). Thus, our results partially support the hypothesis that MPV is related to schizophrenia, and further research is needed to establish whether there is an underlying chronic inflammatory process.

Similarly, NLR has been proposed as a biomarker for schizophrenia and even for other neurodegenerative diseases (23). This is also ratified by this study since the age of symptom onset correlated positively and significantly with NLR, data already reported by other authors, which has been interpreted as indicative of a chronic inflammatory state both in schizophrenia and in bipolar disorder, so it could potentially be used as an indicator of the evolution of psychosis symptoms (24, 25).

The elevation of MPV with respect to healthy subjects has also been observed in subjects with depression, independently of antipsychotic treatment (26). High MPV promotes platelet serotonin release in schizophrenia but not in depression, which is related to disease duration but not to age (21).

As we have mentioned, high MPV is associated with the presence of cardiovascular complications; in our opinion, it could be considered an indicator of cardiovascular risk in this population, i.e., those patients who exceed the MPV cutoff point, yet to be defined, although 8.2 fl has been proposed as a cutoff point (13), would be at greater cardiovascular risk, so there would be clinical elements to differentiate those who require greater cardiovascular follow-up as indicated in clinical practice guidelines.

We emphasize that we included subjects whose laboratory samples were taken under the same conditions of fasting and schedule, who had never taken psychopharmacological treatment, and who did not suffer from any general medical disease either clinically or by the laboratory, aspects not always reported in similar studies. Our study has the limitation that it was neither possible to measure the intensity of the symptoms in the sample, as it was a retrospective study, nor was it possible to directly evaluate the controls. Nor was it possible to directly evaluate the controls to determine substance use or BMI; nevertheless, the results reveal high MPV in the patients in comparison with the controls. In addition, it was not possible to exclude other possible causes of high MPV, such as vitamin D deficiency, treatment with cyanocobalamin to treat vitamin B12 deficiency, or the use of multivitamins.

We think that it is necessary to analyze the conditions under which other studies have been done, for example, the equipment used, normal reference ranges, type of anticoagulant (EDTA or citrate), ethnicity, geographical conditions (altitude) in order to compare the results and be able to get conclusions (2, 27). Even so, our results support the inflammatory hypothesis of schizophrenia, that is, that both MPV and NLR could be biomarkers of clinical utility in patients with schizophrenia. Future studies should establish some cut-off point for MPV and NLR and follow up those patients who exceed them, to determine whether they were at increased cardiovascular risk.

## Data availability statement

The raw data supporting the conclusions of this article will be made available by the authors, without undue reservation.

## Ethics statement

The study was conducted with clinical and laboratory records of patients and controls performed between January 2016 and October 2018, because it was retrospective, informed consent was not used. It was approved by the Ethics Committee of the Psychiatric Hospital “Fray Bernardino Álvarez”.

## Author contributions

HC-R: substantial contributions to the conception and design of the study. MB-M: substantial contributions to the design of the study and analysis of data. EB-A: organization of the database. OP-G: contributions to the acquisition of the database. HC-R and MB-M: drafting of the study. HC-R, MB-M, EB-A, and OP-G: approval for the publication of the content. All authors contributed to the article and approved the submitted version.
